# Molecular basis of hyper-thermostability in the thermophilic archaeal aldolase MfnB

**DOI:** 10.1007/s00792-024-01359-x

**Published:** 2024-08-31

**Authors:** Rosie M. A. Maddock, Carl O. Marsh, Samuel T. Johns, Lynden D. Rooms, Phillip W. Duke, Marc W. van der Kamp, James E. M. Stach, Paul R. Race

**Affiliations:** 1 School of Biochemistry, Biomedical Sciences Building, University of Bristol, University Walk, Bristol, BS8 1TD UK; 2 BrisSynBio Synthetic Biology Research Centre, Life Sciences Building, University of Bristol, Tyndall Avenue, Bristol, BS8 1TQ UK; 3 Defence Science and Technology Laboratory, Porton Down, Salisbury, SP4 0JQ UK; 4 School of Natural and Environmental Sciences, Newcastle University, Newcastle upon Tyne, NE1 7RU UK

**Keywords:** Methanogenesis, Methanofuran, Aldolase, Protein folding, Thermostability

## Abstract

Methanogenic archaea are chemolithotrophic prokaryotes that can reduce carbon dioxide with hydrogen gas to form methane. These microorganisms make a significant contribution to the global carbon cycle, with methanogenic archaea from anoxic environments estimated to contribute > 500 million tons of global methane annually. Archaeal methanogenesis is dependent on the methanofurans; aminomethylfuran containing coenzymes that act as the primary C_1_ acceptor molecule during carbon dioxide fixation. Although the biosynthetic pathway to the methanofurans has been elucidated, structural adaptations which confer thermotolerance to Mfn enzymes from extremophilic archaea are yet to be investigated. Here we focus on the methanofuran biosynthetic enzyme MfnB, which catalyses the condensation of two molecules of glyceralde-3-phosphate to form 4‑(hydroxymethyl)-2-furancarboxaldehyde-phosphate. In this study, MfnB enzymes from the hyperthermophile *Methanocaldococcus jannaschii* and the mesophile *Methanococcus maripaludis* have been recombinantly overexpressed and purified to homogeneity. Thermal unfolding studies, together with steady-state kinetic assays, demonstrate thermoadaptation in the *M. jannaschii* enzyme. Molecular dynamics simulations have been used to provide a structural explanation for the observed properties. These reveal a greater number of side chain interactions in the *M. jannaschii* enzyme, which may confer protection from heating effects by enforcing spatial residue constraints.

**Supplementary Information:**

The online version contains supplementary material available at 10.1007/s00792-024-01359-x.

## Introduction

Methanogenesis is a specialised form of anaerobic respiration which involves the oxidation of hydrogen gas to H^+^ and the concomitant reduction of carbon dioxide, or other one-carbon molecules, to generate methane (Jones et al. 1985; Thauer 1998). This unique metabolic adaptation is found exclusively in the methanogenic archaea, obligate methane producers that do not grow using fermentation or via the use of alternative electron acceptors for respiration (Berghuis et al. 2019). Methanogenic archaea are major contributors to the global carbon cycle and consequently play an important role in climate change (Reeburgh 2007; Thauer et al. 2008; Conrad 2009).

**Fig. 1:**
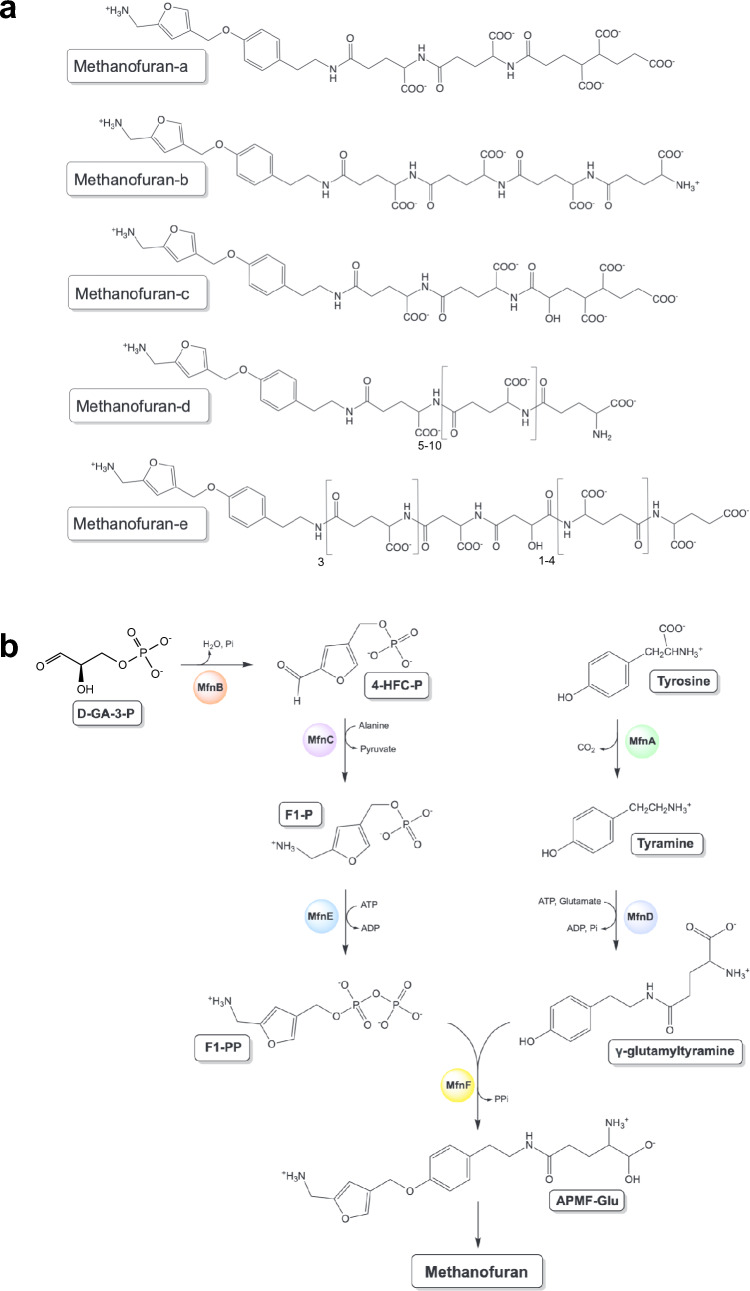
The methanofurans and their biosynthesis. **a** Chemical structures of known methanofuran cofactors from methanogenic archaea. **b** Generalised biosynthetic pathway to the methanofurans. D-GA-3P, D-glyceraldehyde-3-phosphate; 4-HFC-P, 4-(hydroxymethyl)-2-furancarboxaldehyde phosphate; F1-P, [5-(aminomethyl)furan-3-yl]methyl phosphate; F1-PP, [5-(aminomethyl)furan-3-yl]methyl diphosphate; APMF-Glu, 4-[N-(-L-glutamyl)-*p*-(-aminoethyl)phenoxymethyl]-(aminomethyl)furan

The methanofurans (a-e; Fig. 1a) are a family of closely related coenzymes that comprise a 4-[*N*-(γ-l-glutamyl)-*p*-(β-aminoethyl)phenoxymethyl]-2-(aminomethyl)furan (APMF-Glu) core, fused to one of five chemically distinct side chains. These molecules function as the primary C_1_ acceptor in methanogenesis, which starts with the two-electron reduction of carbon dioxide to form formyl-methanofuran (Leigh and Wolfe 1983; Leigh et al. 1984, 1985; White 1988; Allen and White 2014; Wagner et al. 2016). Importantly, methanofuran formylation represents one of the few known biological routes to carbon dioxide fixation (Braakman and Smith 2012). The methanofurans have also been shown to play an important role in methylotrophic bacteria, where they function as a coenzyme in the oxidation of formaldehyde to carbon dioxide (Chistoserdova et al. 1998; Vorholt et al. 1999).

The biosynthetic pathway to the methanofurans has been the subject of considerable investigation, though questions remain regarding the identity of several key pathway intermediates, whose chemical structures are yet to be unambiguously elucidated (Wang et al. 2003, 2014; Kezmarsky et al. 2005; Bobik et al. 2014; Miller et al. 2014; Wang et al. 2015a, b). Similarly, much still remains to be learned about the structures and catalytic mechanisms of the enzymes involved in the assembly of the methanofuran scaffold. In vitro studies using purified recombinant enzymes have demonstrated that methanofuran biosynthesis occurs via a bifurcated pathway, where the enzymes MfnB, C and E construct the 5-(aminomethyl)-3-furanmethanol-phosphate (F1-P) moiety of the molecule, whilst MfnA and D assemble the γ-glutamyltyramine component. These two branches converge at the enzyme MfnF, which couples the two intermediates via an ether bond forming condensation reaction (Fig. 1b).

Here we focus on the methanofuran biosynthetic enzyme MfnB, a type I aldolase that catalyses the condensation of two molecules of glyceraldehyde-3-phosphate (GA-3-P) to form 4-(hydroxymethyl)-2-furancarboxaldehyde-phosphate (4-HFC-P; Fig. 1b). Previous structural and mechanistic studies of this enzyme have focused exclusively on MfnB from the hyperthermophile *M. jannaschii*, a deep-sea methanogenic archaea with an optimal growth temperature of > 85 °C (Jones et al. 1983). The X-ray crystal structure of this enzyme has been determined, which reveals a homohexameric assembly, within which each monomer adopts a classical TIM-barrel fold (Bobik et al. 2014). Complimentary in vitro functional studies, using wild-type and mutant MfnB enzymes, suggests a multi-step ternary complex reaction mechanism, which requires the simultaneous occupation of two distinct GA-3-P binding sites within the enzyme. The molecule bound in site 1 undergoes a phosphate elimination reaction, whereas that in site 2 undergoes a triose phosphate isomerase-like reaction. A subsequent aldol condensation is proposed to take place between the enzyme-bound enol form of methylglyoxal and dihydroxyacetone phosphate (DHAP), followed by product cyclisation (Wang et al. 2015a, b). Significantly, published in vitro enzyme assays of *M. jannaschii* MfnB have been exclusively performed at 70 °C, presumably reflecting the limited capacity of this enzyme to catalyse 4-HFC-P formation at lower temperatures (Wang et al. 2015a, b). This temperature requirement limits the usefulness of *M. jannaschii* MfnB as a biocatalyst for di-substituted furan production, an important consideration given that such enzymes are highly sought after due to the usefulness of furan heterocycles as fuels and materials (Wang et al. 2018).

In an effort to investigate thermoadaptation in MfnB enzymes, we have conducted a thermal denaturation study of *M. jannaschii* MfnB, benchmarking the unfolding behaviour of this polypeptide against the equivalent enzyme from the mesophile *M. maripaludis*. Complimentary steady-state kinetic studies have also been undertaken, which establish the temperature dependence of both the *M. jannaschii* and *M. maripaludis* MfnB catalysed reactions. These studies also reveal that *M. maripaludis* MfnB is stereoselective for the D-isomer of GA-3-P, in contrast to the *M. jannaschii* enzyme, which is found to accept and act upon L-GA-3-P, albeit with significantly inferior kinetic parameters than for the D-isomer. Our kinetic analyses also reveal direct evidence for substrate inhibition in the MfnB catalysed reaction. To rationalise our observed biophysical and kinetic data, we have performed molecular dynamics simulations of both *M. jannaschii* and *M. maripaludis* MfnB. These analyses reveal markedly increased dynamic motions in the *M. maripaludis* enzyme as compared to that from *M. jannaschii*, consistent with reduced thermostability and in accordance with our biophysical data.

## Materials and methods

### Phylogenetic analysis

Gene sequences for *mfn*B were obtained from NCBI and ENA public databases (Kanz et al. 2005; Agarwala et al. 2016). Sequences were selected from representatives of the main orders of the methanogens. Thermotolerance of the cognate MfnB protein was predicted using the PhyMet2 database (Michał et al. 2018), with predictions then ratified by cross-referencing against the paper reporting the initial identification and characterisation of each strain included in our analysis. Full details of the species and sequences used are given in Table S1. DNA sequences were aligned with Clustal Omega (Sievers et al. 2011), edited with GBlocks (Castresana 2000) to remove poorly aligned or divergent regions, and a maximum likelihood tree was constructed with PhyML (Guindon et al. 2010) using a GTR + G + I model and aBayes branch support values. MfnB protein sequence alignments were annotated with ESPript (Robert and Gouet 2014).

### Gene cloning

Genes encoding MfnB from *M. jannaschii* DSM 2661 and *M. maripaludis* str. S2 (Jones et al. 1983), from hereon referred to as MfnB_MJ and MfnB_MM respectively, were PCR amplified from commercially sourced synthetic genes (Thermo Fisher Scientific) codon optimised for expression in *Escherichia coli* (Raab et al. 2010). PCR reactions were conducted using the primers MfnB_MJ_fwd 5′-AAGTTCTGTTTCAGGGCCCGATGATACTATTAGTAAGCCC-3′, MfnB_MJ_rev 3′-ATGGTCTAGAAAGCTTTATTACTTACAAAGCTCCTTTAAC-5′, MfnB_MM_fwd 5′-AAGTTCTGTTTCAGGGCCCGATGATTCTGCTGGTTAGCCCG-3′, and MfnB_MM_rev 3′-ATGGTCTAGAAAGCTTTATTACTGACGGCACACTTTCAC-5′, respectively, which incorporate primer extensions (underlined) to enable cloning into the protein expression vector *p*OPINF (Berrow et al. 2007) using the In-Fusion™ recombinase (Clontech). PCR products were purified and ligated into pre-digested (*Kpn*1 and *Hind*III) *p*OPINF, to yield the plasmids *mfn*B_MJ::*p*OPINF and *mfn*B_MM::*p*OPINF, which encode N-terminally hexa-histidine tagged versions of MfnB_MJ and MfnB_MM respectively. Both constructs were verified by DNA sequencing and subsequently transformed into *E. coli* BL21 (DE3) cells for protein expression.

### Protein expression and purification

*E. coli* BL21 (DE3) cells transformed with *mfn*B_MJ::*p*OPINF or *mfn*B_MM::*p*OPINF were grown in 1 L of Luria–Bertani (LB) medium supplemented with carbenicillin (50 μg/mL) at 37 °C, with shaking at 180 rpm, to an OD_600nm_ of ~ 0.6. Protein expression was induced by the addition of Isopropyl β-D-1-thiogalactopyranoside (IPTG) to a final concentration of 0.1 M. Cell suspensions were subsequently cultured for a further 16 h with shaking at 180 rpm at 20 °C. Cells were harvested by centrifugation at 5,000 × *g* and resulting pellets stored at -80 °C. Cell pellets were thawed on ice, re-suspended in lysis buffer (20 mM Tris–HCl, 150 mM NaCl, 20 mM imidazole, pH 7.5) and lysed using a cell disruptor (Z Plus Series cell disruptor, Constant Systems Ltd.) at 25,000 psi. Cell lysates were clarified by centrifugation at 20,000 × *g* and recovered supernatants applied to a 5 mL His-Trap column (GE Healthcare Life Sciences) pre-loaded with nickel. Hexa-histidine tagged MfnB proteins were eluted using an imidazole gradient of 0.02–1 M. Eluted fractions were analysed by SDS-PAGE and those found to contain protein with a molecular weight equivalent to that predicted for MfnB_MJ or MfnB_MM were pooled, concentrated and subjected to a further purification step via passage through a Superdex 16/600 S200 column (GE Healthcare) pre-equilibrated in 20 mM Tris–HCl, 150 mM NaCl, pH 7.5. Eluted fractions containing the protein of interest were pooled, concentrated to 1 mg/mL, and flash frozen in liquid nitrogen prior to storage at − 80 °C. The estimated purity of both MfnB enzymes was judged to be > 95% as determined by SDS-PAGE analysis. Protein identity was confirmed by mass spectrometry following trypsin digestion. Frozen protein samples were thawed on ice for 30 min prior to further analysis.

### Thermal denaturation studies using circular dichroism spectroscopy

Purified proteins were dialysed into Circular Dichroism (CD) buffer (20 mM sodium phosphate, pH 7.5) for 24 h at 4 °C. Following dialysis protein samples were centrifuged at 14,000 × *g* to remove aggregates. Proteins were diluted in CD buffer to 0.15 mg/mL immediately prior to analysis. CD measurements were performed using a Jasco J-1500 CD spectrometer fitted with a Peltier temperature control unit, with protein samples housed in a 1 mm pathlength quartz cuvette (Hellma Analytics). Thermal denaturation was achieved by heating samples from 25 to 95 °C in 1 °C increments, with continual monitoring of sample ellipticity from 190 to 255 nm throughout. Measurements incorporated a 60 s thermal equilibration step between each data collection point. All spectra were collected in quadruplet and averaged to generate mean ellipticity values. A scanning speed of 100 nm/min was used, with a spectral bandwidth of 2 nm and a data pitch of 1.0 nm. For all experiments a high-tension (HT) voltage of < 700 V was used as the quality cut-off for the CD signal. Generated CD spectra were smoothed using a Savitzky-Golay filter, with fraction folded values calculated from mean sample ellipticity readings at 222 nm. Thermal unfolding profiles were fitted to a Boltzmann sigmoidal equation assuming a two-state model using GraphPad Prism 10.

### Steady-state kinetic characterisation of MfnB enzymes

Steady-state kinetic assays of MfnB_MJ and MfnB_MM were performed using a previously reported spectrophotometric assay, monitoring the MfnB catalysed formation of 4-HFC-P at 280 nm (ε = 15,900 M^−1^ cm^−1^; Wang et al. 2015a, b). Assays were performed in a 1 cm pathlength quartz cuvette (Hellma Analytics) in MfnB reaction buffer (20 mM Tris–HCl, 150 mM NaCl, pH 7.5), with reaction mixes comprising D-GA-3-P and/or L-GA-3-P (0–1 mM) and 4 ug of enzyme. Assays were performed in triplicate, by monitoring the change in absorbance at 280 nm for at least 4 min. At each concentration of substrate, reaction rates recorded in the absence of enzyme were subtracted from those recorded in the presence of enzyme. To establish the temperature dependence of the MfnB catalysed reaction assays were performed at 25—85 °C, with temperature control achieved using a Peltier temperature control unit. All assay components were equilibrated to the required temperature for at least 10 min prior to measurement. For data analysis, plots of initial velocity (*v*_i_) versus substrate concentration [*S*] were fitted globally using GraphPad Prism 10 to the following equation, which accounts for an observed contribution of substrate inhibition:

$${v}_{i}=\frac{{k}_{cat}[E]\left[S\right]}{{K}_{m}+\left[S\right]\left(\frac{1+\left[S\right]}{{K}_{i}}\right)}$$

where [*E*] is enzyme concentration, *k*_cat_ is the turnover number, *K*_m_ is the Michaelis constant, and *K*_i_ is the inhibition constant.

### Molecular dynamics simulations

Molecular dynamics (MD) simulations were performed using the published crystal structure of *M. jannaschii* MfnB (PDB 4RC1; Bobik et al. 2014) and a homology model of the *M. maripaludis* enzyme. The latter was generated using the webserver I-TASSER (Roy et al. 2010) employing chain A from the crystal structure of MfnB_MJ as the template (> 70% sequence identity). The top ranked MfnB_MM model (C-score of 1.87, TM-score of 0.98 ± 0.05, Cα root mean square deviation (RMSD) of 2.1 ± 1.6 Å) was chosen for comparative molecular dynamics simulations with 4RC1 chain A. MfnB_MJ chain A was stripped of its active site bound phosphate ion, as phosphate ions are present in some but not all protomers. Additionally, the N-terminal hexa-histidine tag of the MfnB_MJ crystal structure was removed and capped with an acetate group, which was attached to the N-terminus of the starting methionine residue. An acetate group was similarly attached to the I-TASSER MfnB_MM homology model to ensure equivalence. Any missing atoms from amino acid side chains were rebuilt into the model using PyMol, specifically, those of K198 and R218. This was also performed for the *M. maripaludis* MfnB homology model. Amino acid protonation states were predicted using PROPKA3 (Olsson et al. 2011). Using the tleap from the AMBER18 suite of programs (Case et al. 2005) both monomeric MfnB structures were solvated in a rectangular box of TIP3P water (Mark and Nilsson 2001), extending at least 10 Å from any protein atom. Na^+^ ions were added to neutralise the systems, placed randomly at least 5 Å from any protein atom for each independent simulation. After initial short energy minimization, both systems were briefly heated (after assigning random velocities at 50 K for each simulation) and then allowed to equilibrate for 500 ps at 348 K and 1 atm in the NPT ensemble (using Langevin dynamics with a 1 ps-1collision frequency and the Berendsen barostat with a 1 ps relaxation time for temperature and pressure control, respectively), with Cα atoms constrained to their starting positions (force constant 5 kcal mol^−1^ Å^−2^, coordinates from PDB ID 4RC1 chain A for MfnB_MJ and the homology model for MfnB_MM). After gradual release of the constraints in 5 ps, two independent 100 ns simulations were then conducted at the same temperature (348 K) and pressure using AMBER18’s pmemd.cuda. A 2 fs timestep, SHAKE restraints and periodic boundary conditions were used throughout, with a cut-off for direct-space non-bonded interactions of 8 Å and Particle-mesh Ewald for longer range electrostatic interactions. Root mean square deviation (RMSD, from the starting structures) and root mean square fluctuations (RMSF, after alignment to an average structure from the trajectory) were then performed using the AmberTools18 program cpptraj.

## Results

### Phylogenetic analysis

**Fig. 2:**
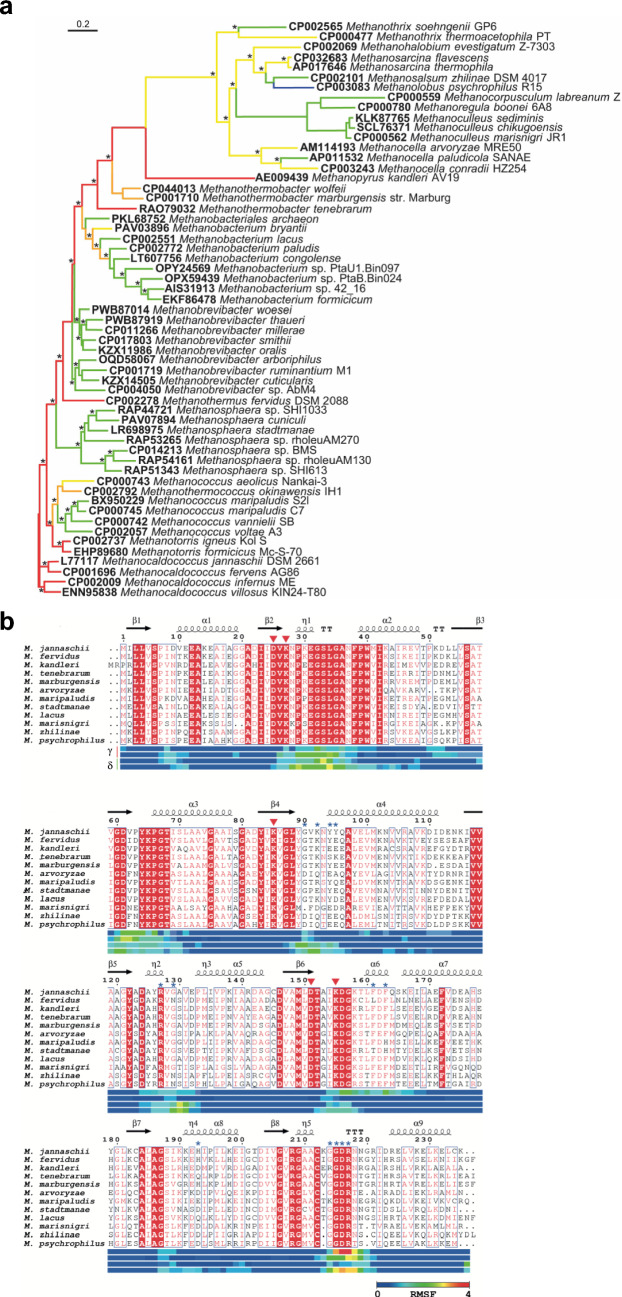
Phylogenetic analysis of MfnB polypeptides. **a** Maximum likelihood phylogenetic tree of *mfn*B sequences. Branches are coloured according to the reported growth characteristics of the species from which the sequences are derived. Red = hyperthermophile, orange = extreme thermophile, yellow = thermophile, green = mesophile, and blue = psychrophile. The alignment was built with Clustal Omega (762 bp) and edited using Gblocks (642 bp). Branch support values > 0.5 are indicated by asterisks. The scale-bar indicates substitutions per site. **b** Amino acid sequence alignment of archaeal MfnB proteins. Sequences are arranged based on predicted thermotolerance (hyperthermophiles top, pyschrophiles bottom). Secondary structure elements in MfnB_MJ are shown above the alignment. Residues coloured white on a red background are conserved in all sequences, those bordered by a blue box possess a physicochemical similarity > 0.7. Residues highlighted by a red triangle have been previously reported to participate in catalysis. Residues highlighted by a blue asterisk are those identified in this study that promote thermotolerance in MfnB_MJ as compared to MfnB_MM (see Figs. 8 and 9 for further details). Horizontal bars below the sequence alignment indicate the RMSF value for each residue from two independent MD simulations of MfnB_MJ (labelled *γ*) and MfnB_MM (labelled *δ*)

To establish the molecular determinants of thermotolerance in MfnB_MJ, we first sought to identify a suitable homologue, from a closely related mesophilic species, for use as a comparator. Sequences of *mfn*B genes from representative species of the main orders of the methanogens were aligned and placed in a maximum likelihood phylogenetic tree (Fig. 2a). From this analysis, the *mfn*B gene of *M. maripaludis* was identified as the mesophilic sequence most closely related to that of the ancestral *M. jannaschii* enzyme. Between MfnB_MM and MfnB_MJ there are 61 amino acid differences from a total of 235 residues (Figs. 2b and S1). Of these differences, 31 (50.8%) result in no change in the charge state of the respective amino acid side chain. Thirteen (21.3%) of these differences introduce hydrophobic amino acids, with 7 (11.5%) introducing positively charged residues and 5 (8.5%) introducing negatively charged residues. Twenty-nine (48.3%) of the 61 differences introduce amino acids with a larger side chain, whilst 22 (36.6%) introduce a residue with a smaller side chain.

### Protein production and characterisation

Codon optimised genes encoding MfnB_MM and MfnB_MJ were successfully cloned into the protein expression vector *p*OPINF and the resulting plasmids used to facilitate the overexpression of recombinant MfnB_MM and MfnB_MJ in *E. coli*. Both proteins were subsequently purified to homogeneity using a combination of nickel affinity chromatography followed by size exclusion chromatography (SEC). Purified recombinant MfnB_MM and MfnB_MJ were both found to be of > 95% purity as established by SDS-PAGE analysis (Figure S2).

### Thermal unfolding studies

**Fig. 3:**
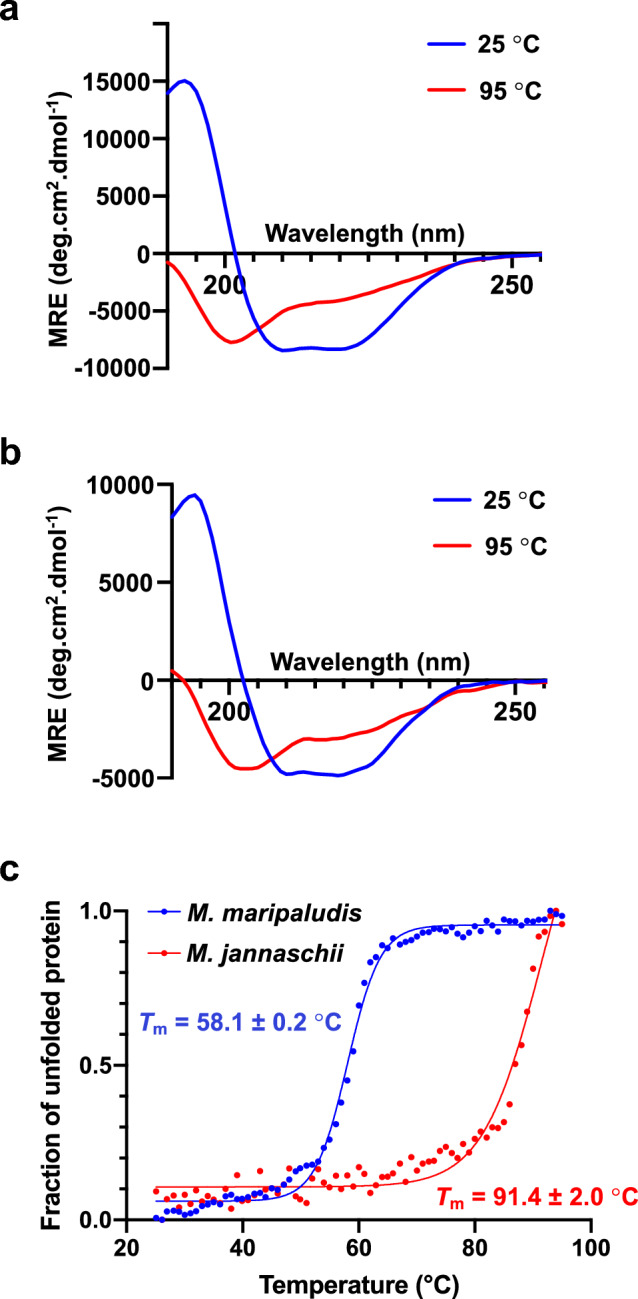
Thermal unfolding studies of MfnB polypeptides. Far-UV CD spectrum of **a** MfnB_MM and **b** MfnB_MJ, at 25 °C and 95 °C. **c** Thermal denaturation of MfnB_MM (blue) and MfnB_MJ (red). Mean residue ellipticity values (MRE) were collected at 222 nm between 25 and 95 °C, in 1 °C increments. Data are fitted to a Boltzmann sigmoidal equation

In an effort to examine thermoadaptation in MfnB polypeptides, the thermal unfolding behaviour of MfnB_MM and MfnB_MJ were investigated using CD spectroscopy. This method enables the quantitation of protein secondary structure composition as a function of temperature. The thermal denaturation of both proteins was investigated by collecting far-UV CD spectra from 190 to 255 nm, following which values for protein melting temperature (*T*_m_) were calculated based on ellipticity measurements at 222 nm. The far-UV CD spectra of MfnB_MM and MfnB_MJ are consistent with both polypeptides being well-folded species in solution, each possessing a mixed α/β structure (Fig. 3a and b). Subsequent thermal denaturation experiments gave unfolding profiles for MfnB_MM and MfnB_MJ consistent with two-state processes, with no observable intermediates (Fig. 3c). As thermal denaturation of both proteins was found to be irreversible, it was not possible to calculate the thermodynamic parameters of unfolding for these polypeptides. However, fitting of the first derivative of each protein’s unfolding profile to a Boltzmann sigmoidal equation enabled elucidation of their respective *T*_m_ values. These were found to be 58.1 ± 0.2 °C for MfnB_MM and 91.4 ± 2.0 °C for MfnB_MJ, consistent with significantly greater thermotolerance in the *M. jannaschii* enzyme as compared to that of *M. maripaludis* (Fig. 3c). It should be noted that our CD data indicate the retention of some secondary structure in MfnB_MJ at 95 °C, the maximum temperature employed in this study (Fig. 3b). Consequently, the calculated *T*_m_ for this polypeptide is likely to represent an underestimate of its true melting temperature.

### Steady-state kinetic studies

**Fig. 4:**
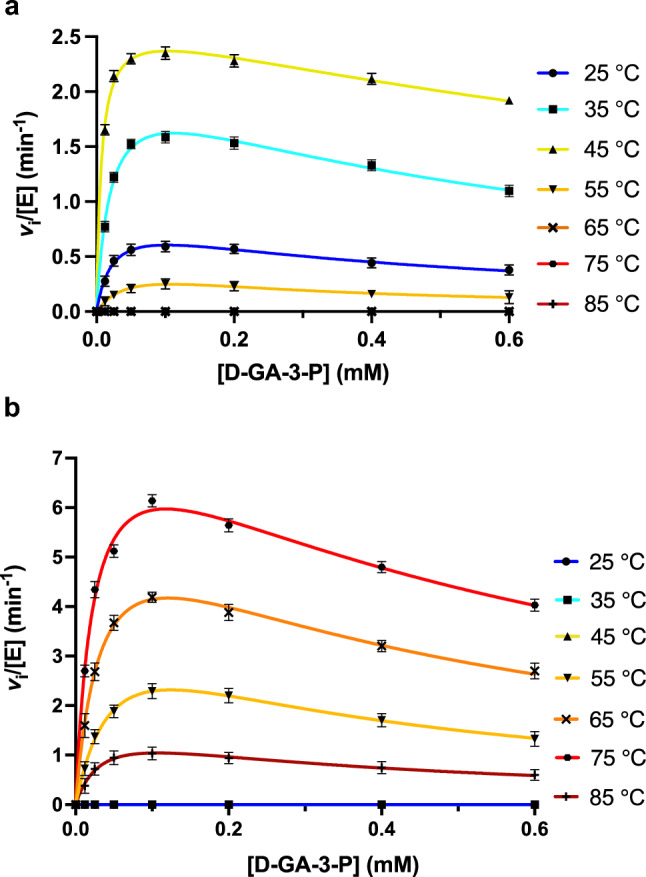
Steady-state kinetic analysis of MfnB polypeptides. Plots of initial velocity (*v*_i_) versus substrate concentration [*S*] for **a** MfnB_MM and **b** MfnB_MJ, for the substrate D-GA-3-P. Data were collected at temperatures ranging from 25 to 85 °C. Data points are mean averages of three repeats of each experiments performed using the same enzyme preparation, with error bars representing the standard errors of the mean. Each curve is fitted independently to a derivative of the Michaelis–Menten equation incorporating a term accounting for substrate inhibition

Following our unfolding studies, we next sought to investigate the effect of temperature on the MfnB catalysed reaction. In vitro steady-state kinetic characterisation of MfnB_MJ and MfnB_MM was performed using a previously reported spectrophotometric assay, monitoring the rate of 4-HFC-P formation from D-GA-3-P (Wang et al. 2015a, b). The temperature dependence of the MfnB_MJ and MfnB_MM catalysed reactions were investigated by performing enzyme assays at temperatures ranging from 25 to 85 °C. Experimentally determined reaction rates (*v*_i_) were subsequently plotted as a function of substrate concentration [*S*] to enable elucidation of the kinetic parameters of both MfnB_MJ and MfnB_MM for D-GA-3-P. Initial inspection of the resulting *v*_i_ vs. [*S*] plots revealed a decrease in reaction rate concomitant with increasing substrate concentration and independent of assay temperature. These data were found to fit convincingly to a derivative of the Michaelis–Menten equation that incorporates a term accounting for substrate inhibition (Fig. 4). From these analyses it was possible to determine values for *k*_cat_, *K*_M_ and *K*_i_, for both MfnB enzymes at the temperatures investigated (Table 1).

**Table 1:** Steady-state kinetic parameters of the MfnB catalysed conversion of D-GA-3-P to 4-HFC-P

Temperature (°C)	Protein (MfnB)	*k*_cat_ (min^−1^)	*K*_M_ (μM)	*k*_cat_/*K*_M_ (μM^−1^.min^−1^)	*K*_i_ (μM)
25	MfnB_MM MfnB_MJ	0.9 ± 0.1 –	23.7 ± 2.8 –	0.04 ± 0.01 –	443.4 ± 54.5 –
35	MfnB_MM MfnB_MJ	2.2 ± 0.1 –	20.4 ± 2.0 –	0.1 ± 0.01 –	620.2 ± 69.1 –
45	MfnB_MM MfnB_MJ	2.7 ± 0.1 –	6.9 ± 0.6 –	0.4 ± 0.04 –	1532.0 ± 160.4 –
55	MfnB_MM MfnB_MJ	0.5 ± 0.1 4.5 ± 0.2	56.7 ± 5.7 57.0 ± 4.8	0.01 ± 0.002 0.08 ± 0.08	207.0 ± 21.2 270.2 ± 23.7
65	MfnB_MM MfnB_MJ	– 6.6 ± 0.3	– 35.2 ± 2.8	– 0.2 ± 0.02	– 414.3 ± 36.5
75	MfnB_MM MfnB_MJ	– 8.5 ± 0.4	– 24.6 ± 3.1	– 0.4 ± 0.08	– 565.3 ± 79.7
85	MfnB_MM MfnB_MJ	– 1.8 ± 0.7	– 37.0 ± 5.6	– 0.05 ± 0.02	– 309.1 ± 47.5

MfnB catalysed reactions were found to exhibit distinct temperature dependencies. The optimum temperature of those tested for MfnB_MJ was found to be 75 °C (*k*_cat_/*K*_M_ = 0.4 ± 0.08 μM^−1^.min^−1^), with the enzyme having no detectable activity in our spectrophotometric assay at temperatures of 45 °C or less. A decrease in MfnB_MJ catalytic competency is seen at temperatures > 75 °C, consistent with our folding studies, which suggest partial denaturation of the polypeptide at temperatures > 80 °C. In contrast, MfnB_MM is optimally active at 45 °C (*k*_cat_/*K*_M_ = 0.4 ± 0.04 μM^−1^.min^−1^) and is capable of catalysing D-GA-3-P condensation at ambient temperature. MfnB_MM exhibits a loss in catalytic competency at temperatures > 45 °C, with no evidence of activity in assays conducted at > 55 °C. These findings are in accord with our thermal denaturation studies of this enzyme, which indicate that it is only partially folded at temperatures > 50 °C.

### Stereoselectivity of the MfnB catalysed reaction

**Fig. 5:**
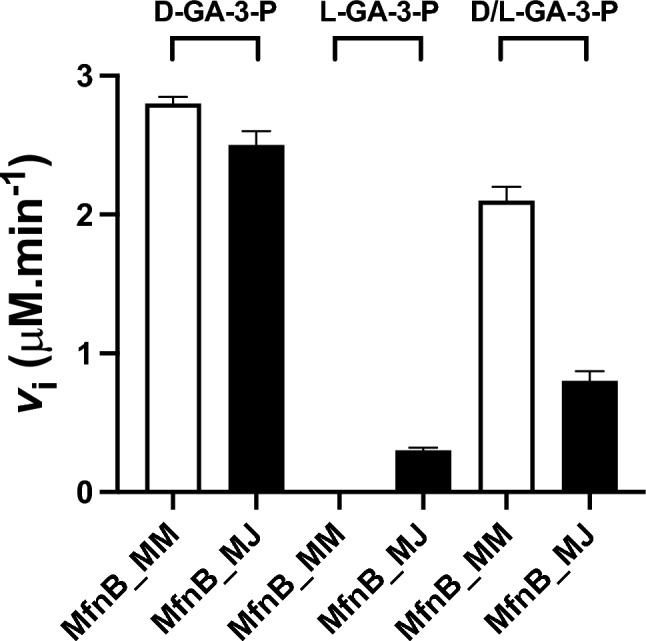
Stereoselectivity of MfnB_MJ and MfnB_MM catalysed reactions. Activity assays monitoring MfnB catalysed formation of 4-HFC-P from D-GA-3-P, L-GA-3-P and 50:50 D/L-GA-3-P. Error bars represent standard errors calculated from triplicate experiments using the same enzyme preparation. Data were collected at 45 °C for MfnB_MM and 75 °C for MfnB_MJ

Having established the temperature dependence of the MfnB_MM and MfnB_MJ catalysed reactions, we next sought to investigate the stereoselectivity of these two biocatalysts. Kinetic assays were performed for both MfnB_MM and MfnB_MJ using either D-GA-3-P, L-GA-3-P, or a 50:50 racemic mixture of D/L-GA-3-P as substrates. Assays were undertaken at a single concentration of each substrate (1 mM; >  × 10 km), at either 45 °C (MfnB_MM) or 75 °C (MfnB_MJ). Assays were performed as outlined previously, monitoring 4-HFC-P formation spectrophotometrically at 280 nm. Surprisingly, MfnB_MJ was found to catalyse the conversion of both the D*-* and L-isomers of GA-3-P to 4-HFC-P, albeit with a clear preference for D-GA-3-P (Fig. 5). Interestingly, in assays incorporating a 50:50 racemic mixture of D/L-GA-3-P, recorded reaction rates were > 50% less than those for D-GA-3-P alone, despite the use of saturating concentrations of substrate. This finding is consistent with L*-*GA-3-P functioning as a competitive inhibitor of the D*-*GA-3-P reaction. In contrast, MfnB_MM was found to accept and act upon D*-*GA-3-P exclusively, with no activity observed for reactions incorporating L*-*GA-3-P as the substrate, and only a modest reduction in the MfnB_MM reaction rate observed in assays incorporating 50:50 D/L-GA-3-P.

### Homology modelling and structural analysis

**Fig. 6:**
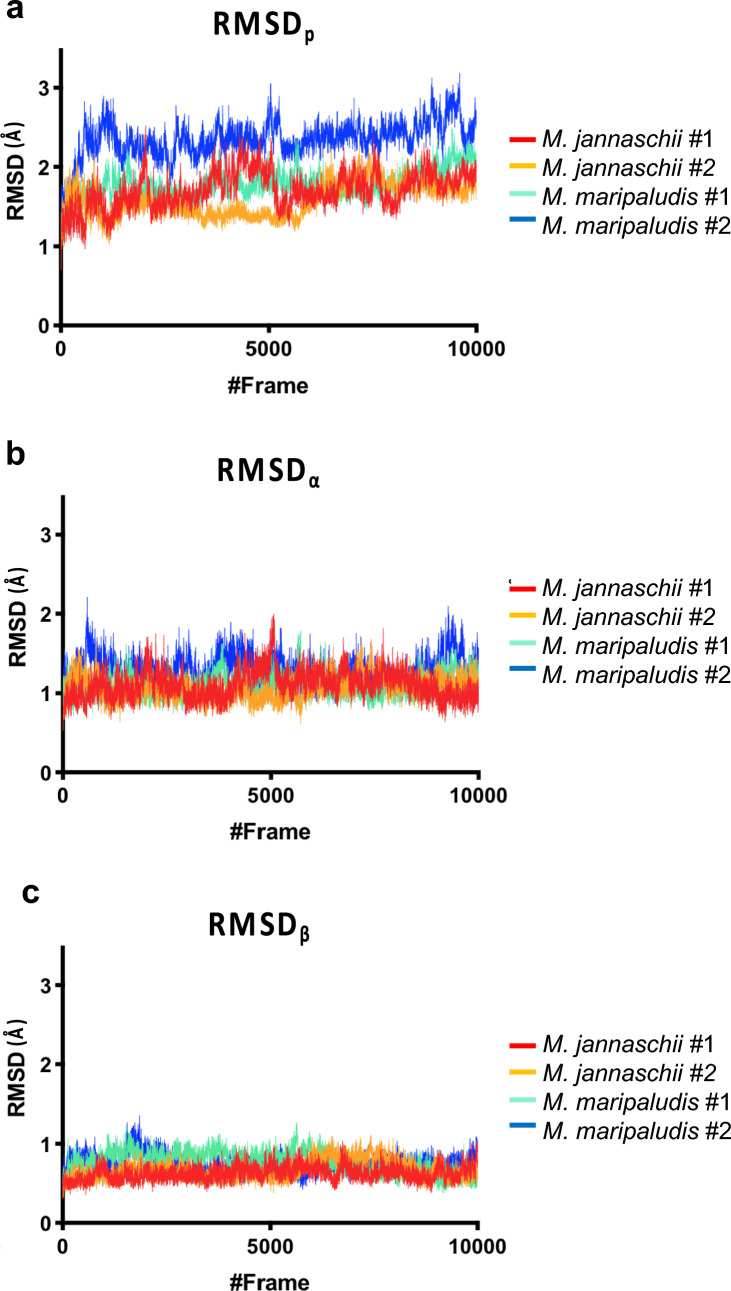
RMSD calculations for MfnB polypeptides. Calculations are shown for **a** RMSDp, **b** RMSDα, and **c** RMSDβ. Data shown are derived from two MD simulations each of MfnB_MJ (red and orange) and MfnB_MM (cyan and blue). Simulations were performed at 75 °C

To provide a structural explanation for our biophysical and kinetic data, we next performed MD simulations of both MfnB_MM and MfnB_MJ. Residue differences identified in our sequence alignment were examined in the context of the crystal structure of MfnB_MJ (PDB 4RC1; Bobik et al. 2014) and a homology model of MfnB_MM. Two independent 100 ns MD simulations were performed to calculate RMSD and RMSF values for both polypeptides. Throughout the simulations, there is less structural change observed in MfnB_MJ than in MfnB_MM, with Cα RMSD values generally being lower (Fig. 6). The average RMSDp values for 10—100 ns of the simulations (1.66 Å and 2.09 Å for the *M. jannaschii* and *M. maripaludis* enzymes, respectively) indicate an increased overall structural change for MfnB_MM (by 23%). This change is also evident when only the beta-barrel is considered (increase in average RMSDβ of 15%), indicating an increased overall stability of the protein fold in MfnB_MJ.

**Fig. 7:**
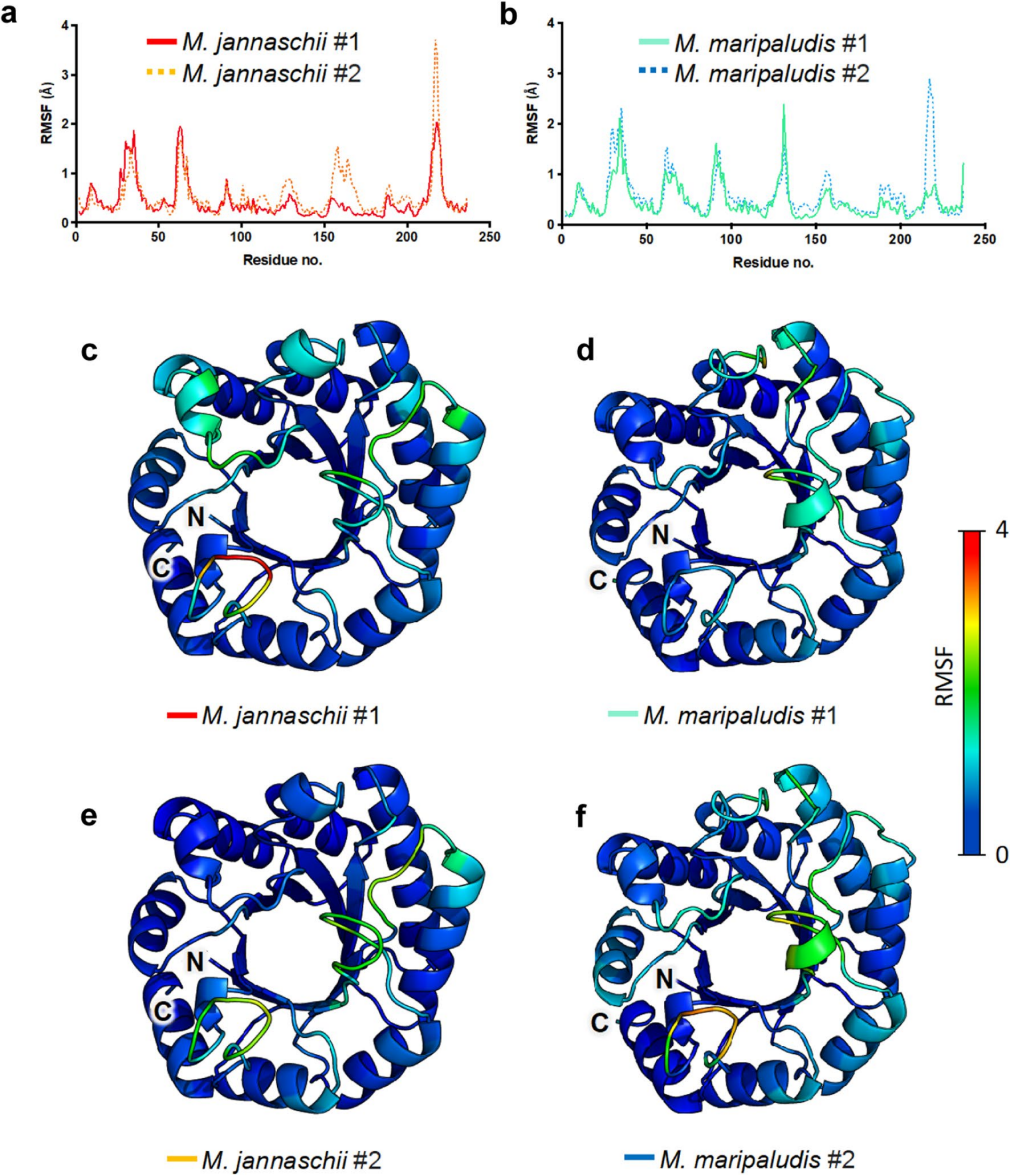
RMSF analysis of MfnB polypeptides. RMSF values for residues in **a** MfnB_MJ and **b** MfnB_MM. RMSF values mapped on the crystal structure of MfnB_MJ from **c** the first simulation and **e** the second simulation. RMSF values mapped on the homology model of MfnB_MM from **d** the first simulation and **f** the second simulation. All values were calculated from MD simulations performed at 75 °C

**Fig. 8:**
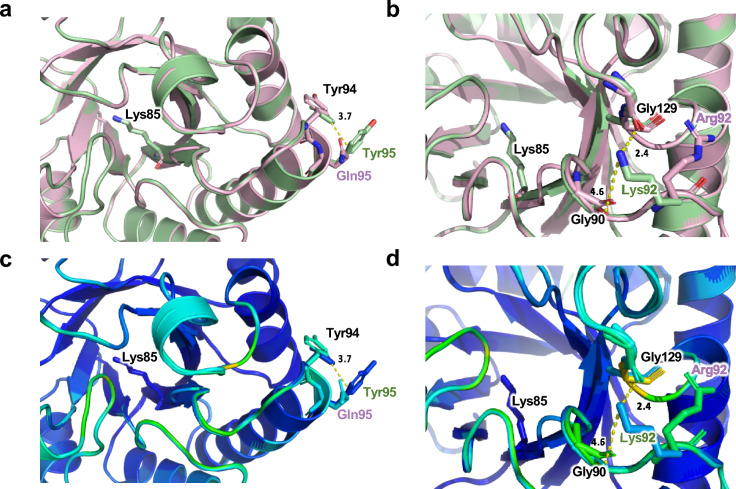
Stabilising interactions in MfnB polypeptides. **a** Superimposition of MfnB_MM (pink) and MfnB_MJ (green), highlighting the Tyr94-Tyr95 helix stabilising interaction in MfnB_MJ. **b** Superimposition of MfnB_MM (pink) and MfnB_MJ (green), highlighting the helix stabilising interaction of Lys92 in MfnB_MJ. **c** and **d** show reproductions of the images shown in (**a**) and (**b**) respectively, coloured by RMSF, from least dynamic (dark blue) to most dynamic (red). Residue labels in black indicate amino acids that are conserved in both MfnB_MM and MfnB_MJ; residue labels in pink indicate amino acids in MfnB_MM only; residue labels in green indicate amino acids in MfnB_MJ only

To account for dynamic motion in loop regions and individual residues, RMSF values for atomic positions were calculated for both MfnB polypeptides (Fig. 7). RMSF analysis indicates region specific differences between the two proteins. For example, although both MfnB_MJ and MfnB_MM exhibit almost identical RMSF values for amino acids 1–90, significant differences are seen in regions spanning residues 90–100 and 130–140. In these regions, MfnB_MM exhibits conformational heterogeneity and a propensity to unfold. In contrast, MfnB_MJ maintains a lower RMSF, consistent with fewer and less pronounced dynamic motions. RMSF values mapped onto the structural models of both proteins are consistent with loop regions, and the termini of helices, exhibiting significant differences in flexibility, suggesting the origins of unfolding propagation (Fig. 7). Superimposition of RMSF values onto our comparative MfnB_MM*/*MfnB_MJ sequence alignment (Fig. 2b), in combination with structural analysis (Fig. 8), provides further insight into the probable molecular basis of thermotolerance in MfnB_MJ as compared to MfnB_MM. For example, R92 in MfnB_MM adopts a rotamer conformation that precludes hydrogen bonding to neighbouring residues. In contrast, the equivalent K92 residue in MfnB_MJ is positioned 2.4 and 4.6 Å respectively from the carbonyls of G129 and G90, with an orientation that favours participation in local bonding interactions (Fig. 8b and d). Interestingly, R92 and Q95 in MfnB_MM, as compared to K92 and Y95 in MfnB_MJ, result in a significant enhancement in local protein mobility, which translates to the largest regional RMSF difference between the two polypeptides over the 100 ns timeframe of the simulation.

**Fig. 9:**
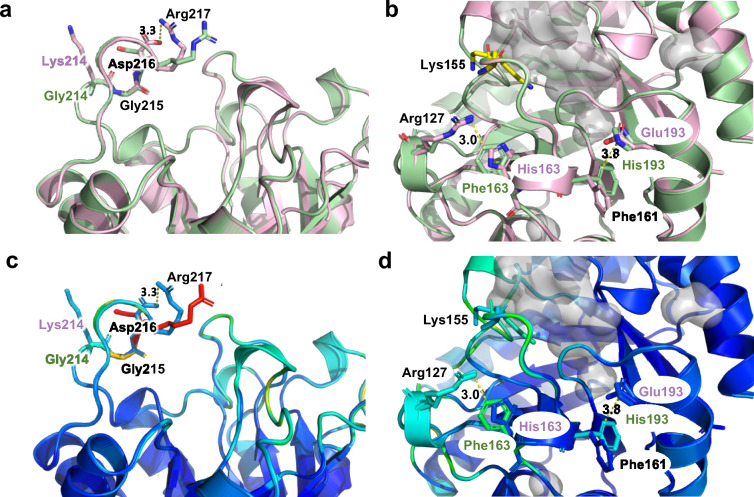
Comparative dynamic motions in MfnB loop regions. **a** Superimposition of MfnB_MJ (green) and MfnB_MM (pink), highlighting flexibility in the active site capping loop. **b** Superimposition of MfnB_MJ (green) and MfnB_MM (pink), highlighting stabilising interactions that influence dynamic motions in the loop housing the catalytic residue Lys155. **c** and **d** show reproductions of the images shown in (**a**) and (**b**) respectively, coloured by RMSF, from least dynamic (dark blue) to most dynamic (red). Residue labels in black indicate amino acids that are conserved in both MfnB_MM and MfnB_MJ; residue labels in pink indicate amino acids in MfnB_MM only; residue labels in green indicate amino acids in MfnB_MJ only

In both MfnB_MM and MfnB_MJ the conserved residue R217 was found to have the highest RMSF. This residue is located on a flexible loop formed by amino acids 213–220, which is situated above the central barrel cavity in both enzymes (Fig. 9). TKGDRNEG in MfnB_MM and KGGDRNNG in MfnB_MJ differ at residues 213 (T to K), 214 (K to G) and 219 (E to N). Interestingly, the glycine pair of KGG in MfnB_MJ appears to function to promote loop destabilisation and enhance flexibility, resulting in a higher RMSF for this residue than is seen in MfnB_MM. Consequently, it appears likely that dynamic motions in this region have little influence on defining global protein thermostability.

## Discussion

The methanofurans are aminomethylfuran containing coenzymes that function as the primary C_1_ acceptor molecule during the biochemical reduction of carbon dioxide to methane in methanogenic archaea. The biosynthesis of these compounds incorporates the type 1 aldolase MfnB, which catalyses the condensation of two molecules of GA-3-P to form 4-HFC-P, a key intermediate on the pathway to this important family of cofactors. In this study a combination of phylogenetic analysis, comparative biophysical and kinetic studies, and molecular dynamics simulations, have been used to investigate the molecular basis of thermoadaptation in MfnB polypeptides.

To date, studies of MfnB have focused exclusively on a species-specific form of this enzyme from the hyperthermophile *M. jannaschii*. To identify a suitable MfnB homologue of mesophilic origin for comparative study, a phylogenetic analysis was performed, which sought to identify the most closely related enzyme of mesophilic origin to MfnB_MJ. Based on this analysis, *M. maripaludis* MfnB was selected as the optimal candidate for further characterisation. Both MfnB_MM and MfnB_MJ were subsequently successfully overexpressed in recombinant form in *E. coli* cells and purified to homogeneity, enabling in vitro characterisation to be undertaken.

To investigate the thermotolerance of purified recombinant MfnB_MJ and MfnB_MM, both polypeptides were subjected to in vitro unfolding analysis. Thermal denaturation of both MfnB_MM and MfnB_MJ were found to be irreversible two-state unfolding processes, with no intermediate species observed. These experiments reveal a significant disparity in the thermotolerance of these two biocatalysts, consistent with their respective thermophilic and mesophilic origins. MfnB_MJ exhibits a melting point temperature (*T*_m_) of 91.4 ± 2.0 °C, as compared 58.1 ± 0.2 °C for MfnB_MM. Notably, for the former polypeptide, there is clear evidence of the retention of secondary structure at temperatures up to and including 95 °C, consistent with the known optimal growth conditions of *M. jannaschii* (Jones et al. 1983).

To expand upon our folding studies, the impact of thermoadaptation on MfnB catalysis was also investigated. Steady-state kinetic assays incorporating MfnB_MM or MfnB_MJ were conducted at a range of temperatures, enabling the elucidation of each enzyme’s respective kinetic parameters for the substrate D-GA-3-P. For both MfnB_MM and MfnB_MJ there was unequivocal evidence of substrate inhibition, a probable consequence of the high proportion of positively charged residues that populate the periphery of each enzyme’s active site, inviting unproductive substrate binding and frustration of the chemical step. Of the temperatures investigated, MfnB_MJ was found to be most active at 75 °C, with a specificity constant (*k*_cat_/*K*_m_) of 0.4 ± 0.08 μM^−1^.min^−1^. In comparison, MfnB_MM was optimally active at 45 °C (*k*_cat_/*K*_m_ = 0.4 ± 0.04 μM^−1^.min^−1^), exhibiting a rapid loss in catalytic activity at temperatures exceeding this. At their respective temperature optima, both enzymes exhibit equivalent *k*_cat_/*K*_m_ values for D-GA-3-P. However, the > twofold enhancement in *k*_cat_ observed in MfnB_MJ, as compared to MfnB_MM, demonstrates superior catalytic proficiency in this biocatalyst, a probable consequence of enhanced dynamic motions promoted by increased temperature. Although our data are consistent with both enzymes employing a common catalytic mechanism, the ability of MfnB_MJ to accept and act upon both D*-* and L-isomers of GA-3-P, and for the later to function as a competitive inhibitor of the former, in contrast to the exclusive D*-*GA-3-P stereoselectivity of MfnB_MM, implies deviation in the precise binding modes of GA-3-P in these two polypeptides.

Differences in thermostability and catalytic behaviour between MfnB_MJ and MfnB_MM arise as a consequence of 61 amino acid differences between the two polypeptides. A comparative sequence alignment reveals a clear bias toward the occupancy of these positions in MfnB_MJ by hydrophobic and positively charged residues (Figs. 2b and S1). Several studies present evidence that support a role for hydrophobic amino acids in excluding buried water molecules and promoting protein stability through structural compaction (Frosst et al. 2000). In contrast, others propose an integral role for buried water molecules in promoting protein stability, at least at ambient temperature, by facilitating the formation of inter-residue hydrogen bonds (Rahaman et al. 2013). Results from our MD simulations indicate that hydrophobic amino acid substitutions in MfnB_MJ may function synergistically to stabilise hydrophobic interfaces between helices, and as a consequence function to promote global rigidity of the protein fold. The acquisition of charged residues that enable salt bridge formation has also been shown to promote protein stability and thermotolerance (Kumar et al. 2002). The increased frequency of residues of this type in MfnB_MJ as compared to MfnB MM is consistent with this hypothesis, though the explicit contributions of individual amino acids can only be formally confirmed using mutagenesis studies.

Our MD simulations reveal large RMSF differences localised to the termini of helices in MfnB polypeptides. Amino acid substitutions in these regions appear to promote more, and stronger bonding interactions in MfnB_MJ as compared to MfnB_MM. For example, Y94 and Y95 are positioned to form a stabilising interaction in MfnB_MJ, in close proximity to K92 (Fig. 8). This latter residue resides in a VKN motif in this enzyme of thermophilic origin. In contrast, mesophilic MfnB_MM possesses a TRS motif in this position, with R92 adopting a rotamer pose precluding hydrogen bond formation with the backbone carbonyl of G129 (Fig. 8). The dihedral angles of the VKN motif of MfnB_MJ are consistent with a K92-G129 interaction, which if present could provide a stabilising effect for the protection of the helix termini and subsequent promotion of structural integrity.

Due to their structural irregularity and inherent flexibility, amino acids that reside within loop regions in proteins are considered to make modest contributions to thermoadaptation. Our MD simulations reveal that in both MfnB_MJ and MfnB_MM, R217, located on a loop region that caps the enzyme active site, has the highest average RMSF (Fig. 7). The positioning of this residue, in combination with its conformational heterogeneity, indicates a possible role in substrate binding, with the guanidinium moiety providing the complimentary positive charge required for interaction with the phosphate group of GA-3-P. The enhanced side chain flexibility of R217 in MfnB_MJ as compared to MfnB_MM, arises as a consequence of increased local conformational dynamics, driven by the presence of a glycine residue at position 214 (Fig. 9a and c). A second prominent example of loop destabilisation in MfnB_MJ is seen in the region housing the substrate binding residue K155 (Fig. 9b and d; Wang et al. 2015a, b). At lower temperatures cation-pi interactions between the residues R127-F163 and F161-H193 may serve to minimise dynamic motions in this region, however, upon heating, perturbation of these stabilising electrostatic interactions could promote flexibility, and facilitate access to the enzyme active site. In MfnB_MM, the equivalent residues R127 and H163, and to a lesser extent F161 and E193, promote inter-residue repulsion, which may account for the observed catalytic activity at ambient temperature.

In summary, here we report comparative biophysical, kinetic and structural analyses of MfnB polypeptides from a hyperthermophilic archaea and a mesophilic archaea. Our findings provide unequivocal evidence of thermoadaptation in the former, despite sharing > 70% amino acid sequence identity with the latter. MD simulations indicate how amino acid substitutions in MfnB_MJ may function to minimise local and global dynamic motions, promoting stability and rigidifying the protein scaffold. These effects are achieved predominately through the stabilisation of inter-helix interfaces resulting from the acquisition of hydrophobic amino acids in these regions, and through the introduction of charged residues that may participate in salt bridges. Our findings provide fundamental insight into thermoadaptation in archaeal proteins and are of value in informing future studies focused on enhancing thermostability in MfnB polypeptides and related carbon–carbon bond forming enzymes. Our study also demonstrates the power of using MD simulations to infer the molecular basis of thermoadaptation in proteins, specifically in cases where the origins of thermotolerance cannot be easily established from comparative amino acid sequences analysis.

## Supplementary Information

Below is the link to the electronic supplementary material.

Supplementary file1 (DOCX 374 KB)

## Data Availability

All data supporting the findings of this study are available within the paper and its Supplementary Information.

## Abbreviations

GA-3-P: Glyceraldehyde-3-phosphate
4-HFC-P: 4-(Hydroxymethyl)-2-furancarboxaldehyde-phosphate
RMSD: Root mean square deviation
RMSF: Root mean square fluctuation
SEC: Size exclusion chromatography
MD: Molecular dynamics

## References

[CR1] Agarwala R, Barrett T, Beck J, Benson DA, Bollin C, Bolton E, Bourexis D et al (2016) Database resources of the national center for biotechnology information. Nucleic Acids Res 44(D1):D7-19. 10.1093/nar/gkv1290 26615191 10.1093/nar/gkv1290 PMC4702911

[CR2] Allen KD, White RH (2014) Identification of structurally diverse methanofuran coenzymes in methanococcales that are both N-formylated and N-acetylated. Biochemistry 53(39):6199–6210. 10.1021/bi500973h 25203397 10.1021/bi500973h

[CR3] Berghuis BA, Feiqiao Brian Yu, Schulz F, Blainey PC, Woyke T, Quake SR (2019) Hydrogenotrophic methanogenesis in archaeal phylum verstraetearchaeota reveals the shared ancestry of all methanogens. Proc Natl Acad Sci USA 116(11):5037–5044. 10.1073/pnas.1815631116 30814220 10.1073/pnas.1815631116 PMC6421429

[CR4] Berrow NS, Alderton D, Sainsbury S, Nettleship J, Assenberg R, Rahman N, Stuart DI, Owens RJ (2007) A versatile ligation-independent cloning method suitable for high-throughput expression screening applications. Nucleic Acids Res. 10.1093/nar/gkm047 17317681 10.1093/nar/gkm047 PMC1874605

[CR5] Bobik TA, Morales EJ, Shin A, Cascio D, Sawaya MR, Arbing M, Yeates TO, Rasche ME (2014) Structure of the methanofuran/methanopterin-biosynthetic enzyme MJ1099 from *Methanocaldococcus Jannaschii*. Acta Crystallogr Sect F: Struct Biol Commun 70(September):1472–79. 10.1107/S2053230X1402130X 25372812 10.1107/S2053230X1402130X PMC4231847

[CR6] Braakman R, Smith E (2012) The emergence and early evolution of biological carbon-fixation. PLoS Comput Biol. 10.1371/journal.pcbi.1002455 22536150 10.1371/journal.pcbi.1002455 PMC3334880

[CR7] Case DA, Cheatham TE, Darden T, Gohlke H, Luo R, Merz KM, Onufriev A, Simmerling C, Wang B, Woods RJ (2005) The amber biomolecular simulation programs. J Comput Chem 26(16):1668–1688. 10.1002/jcc.20290 16200636 10.1002/jcc.20290 PMC1989667

[CR8] Castresana J (2000) Selection of conserved blocks from multiple alignments for their use in phylogenetic analysis. Mol Biol Evol 17(4):540–552. 10.1093/oxfordjournals.molbev.a026334 10742046 10.1093/oxfordjournals.molbev.a026334

[CR9] Chistoserdova L, Vorholt JA, Thauer RK, Lidstrom ME (1998) C1 transfer enzymes and coenzymes linking methylotrophic bacteria and methanogenic archaea. Science 281(5373):99–102. 10.1126/science.281.5373.99 9651254 10.1126/science.281.5373.99

[CR10] Conrad R (2009) The global methane cycle: recent advances in understanding the microbial processes involved. Environ Microbiol Rep 1(5):285–292. 10.1111/j.1758-2229.2009.00038.x 23765881 10.1111/j.1758-2229.2009.00038.x

[CR11] Frosst P, Blom HJ, Milos R, Goyette P, Sheppard CA, Matthews RG, Boers GJ et al (2000) Factors enhancing protein thermostability. Protein Eng Des Sel 13(3):179–191. 10.1038/ng0595-111

[CR12] Guindon S, Dufayard JF, Lefort V, Anisimova M, Hordijk W, Gascuel O (2010) New algorithms and methods to estimate maximum-likelihood phylogenies: assessing the performance of PhyML 3.0. Syst Biol 59(3):307–321. 10.1093/sysbio/syq010 20525638 10.1093/sysbio/syq010

[CR13] Haines NR, VanZanten AN, Cuneo AA, Miller JR, Andrews WJ, Carlson DA, Harrington RM et al (2011) A sulfone-based strategy for the preparation of 2,4-disubstituted furan derivatives. J Org Chem 76(19):8131–8137. 10.1021/jo201529s 21854041 10.1021/jo201529s

[CR14] Jones WJ, Leigh JA, Mayer F, Woese CR, Wolfe RS (1983) *Methanococcus Jannaschii* Sp. Nov., an extremely thermophilic methanogen from a submarine hydrothermal vent. Arch Microbiol 136(4):254–261. 10.1007/BF00425213

[CR15] Jones WJ, Donnelly MI, Wolfe RS (1985) Evidence of a common pathway of carbon dioxide reduction to methane in methanogens. J Bacteriol 163(1):126–131. 10.1128/jb.163.1.126-131.1985 3924891 10.1128/jb.163.1.126-131.1985 PMC219089

[CR16] Kanz C, Aldebert P, Althorpe N, Baker W, Baldwin A, Bates K, Browne P et al (2005) The EMBL nucleotide sequence database. Nucleic Acids Res 33(DATABASE ISS):29–33. 10.1093/nar/gki098 10.1093/nar/gki098 PMC540052 15608199

[CR17] Kezmarsky ND, Huimin Xu, Graham DE, White RH (2005) Identification and characterization of a L-tyrosine decarboxylase in *Methanocaldococcus Jannaschii*. Biochim Biophys Acta Gen Subj 1722(2):175–182. 10.1016/j.bbagen.2004.12.003 10.1016/j.bbagen.2004.12.003 15715981

[CR18] Kramer S, Skrydstrup T (2012) Gold-catalyzed carbene transfer to alkynes: access to 2,4-disubstituted furans. Angew Chem-Int Edition 51(19):4681–4684. 10.1002/anie.201200307 10.1002/anie.201200307 22528137

[CR19] Kumar S, Tsai C-J, Nussinov R (2002) Factors enhancing protein thermostability. Protein Eng Des Sel 13(3):179–191. 10.1093/protein/13.3.179 10.1093/protein/13.3.179 10775659

[CR20] Leigh JA, Wolfe RS (1983) Carbon dioxide reduction factor and methanopterin, two coenzymes required for CO_2_ reduction to methane by extracts of methanobacterium. J Biol Chem 258(12):7536–7540. 10.1016/s0021-9258(18)32210-5 6408076

[CR21] Leigh JA, Rinehart KL, Wolfe RS (1984) Structure of methanofuran, the carbon dioxide reduction factor of methanobacterium thermoautotrophicum. J Am Chem Soc 106(10):3636–3640. 10.1021/ja00324a037

[CR22] Leigh JA, Rinehart KL, Wolfe RS (1985) Methanofuran (carbon dioxide reduction factor), a formyl carrier in methane production from carbon dioxide in methanobacterium. Biochemistry 24(4):995–999. 10.1021/bi00325a028 3922409 10.1021/bi00325a028

[CR23] Mark P, Nilsson L (2001) Structure and dynamics of the TIP3P, SPC, and SPC/E water models at 298 K. J Phys Chem A 105(43):9954–9960. 10.1021/jp003020w

[CR24] Michał B, Gagat P, Jabłoński S, Chilimoniuk J, Gaworski M, Mackiewicz P, Marcin Ł (2018) PhyMet2: a database and toolkit for phylogenetic and metabolic analyses of methanogens. Environ Microbiol Rep 10(3):378–382. 10.1111/1758-2229.12648 29624889 10.1111/1758-2229.12648

[CR25] Miller D, Wang Yu, Huimin Xu, Harich K, White RH (2014) Biosynthesis of the 5-(Aminomethyl)-3-furanmethanol moiety of methanofuran. Biochemistry 53(28):4635–4647. 10.1021/bi500615p 24977328 10.1021/bi500615p

[CR26] Olsson MHM, Søndergaard CR, Rostkowski M, Jensen JH (2011) PROPKA3: consistent treatment of internal and surface residues in empirical PKa predictions. J Chem Theory Comput 7(2):525–537. 10.1021/ct100578z 26596171 10.1021/ct100578z

[CR27] Raab D, Graf M, Notka F, Schödl T, Wagner R (2010) The Gene optimizer algorithm : using a sliding window approach to cope with the vast sequence space in multiparameter DNA sequence optimization. Syst Synth Biol. 10.1007/s11693-010-9062-3 21189842 10.1007/s11693-010-9062-3 PMC2955205

[CR28] Rahaman O, Melchionna S, Laage D, Sterpone F (2013) The effect of protein composition on hydration dynamics. Phys Chem Chem Phys 15(10):3570–3576. 10.1039/c3cp44582h 23381660 10.1039/C3CP44582H PMC3827537

[CR29] Reeburgh WS (2007) Oceanic methane biogeochemistry. Chem Rev 107(2):486–513. 10.1021/cr050362v 17261072 10.1021/cr050362v

[CR30] Robert X, Gouet P (2014) Deciphering key features in protein structures with the new ENDscript server. Nucleic Acids Res 42(W1):320–324. 10.1093/nar/gku316 10.1093/nar/gku316 PMC4086106 24753421

[CR31] Roy A, Kucukural A, Zhang Y (2010) I-TASSER: a unified platform for automated protein structure and function prediction. Nat Protoc 5(4):725–738. 10.1038/nprot.2010.5 20360767 10.1038/nprot.2010.5 PMC2849174

[CR32] Sievers F, Wilm A, Dineen D, Gibson TJ, Karplus K, Li W, Lopez R et al (2011) Fast, scalable generation of high-quality protein multiple sequence alignments using clustal omega. Mol Syst Biol. 10.1038/msb.2011.75 21988835 10.1038/msb.2011.75 PMC3261699

[CR33] Thauer RK (1998) Biochemistry of methanogenesis : a tribute to marjory stephenson. Microbiology. 10.1099/00221287-144-9-2377 9782487 10.1099/00221287-144-9-2377

[CR34] Thauer RK, Kaster A-K, Seedorf H, Buckel W, Hedderich R (2008) Methanogenic archaea: ecologically relevant differences in energy conservation. Nat Rev Microbiol 6(8):579–591. 10.1038/nrmicro1931 18587410 10.1038/nrmicro1931

[CR35] Vorholt JA, Chistoserdova L, Stolyar SM, Thauer RK, Lidstrom ME (1999) Distribution of tetrahydromethanopterin-dependent enzymes in methylotrophic bacteria and phylogeny of methenyl tetrahydromethanopterin cyclohydrolases. J Bacteriol 181(18):5750–5757. 10.1128/jb.181.18.5750-5757.1999 10482517 10.1128/jb.181.18.5750-5757.1999 PMC94096

[CR36] Wagner T, Ermler U, Shima S (2016) The Methanogenic CO2 reducing-and-fixing enzyme is bifunctional and contains 46 [4Fe-4S] clusters. Science 354(6308):114–117. 10.1126/science.aaf9284 27846502 10.1126/science.aaf9284

[CR37] Wang X, Yuan Ye, Teng M, Niu L, Gao Y, Yang JK, Chang C et al (2003) Crystallization and preliminary X-ray analysis of the Mj0684 gene product, a putative aspartate aminotransferase, from *Methanococcus Jannaschii*. Acta Crystallogr, Sect f: Struct Biol Cryst Commun 59(11):563–565. 10.1107/S0907444903000076 10.1107/s0907444903000076 12595727

[CR38] Wang Yu, Huimin Xu, Harich KC, White RH (2014) Identification and characterization of a tyramine-glutamate ligase (MfnD) involved in methanofuran biosynthesis. Biochemistry 53(39):6220–6230. 10.1021/bi500879h 25211225 10.1021/bi500879h

[CR39] Wang Yu, Jones MK, Huimin Xu, Keith Ray W, White RH (2015a) Mechanism of the enzymatic synthesis of 4-(Hydroxymethyl)-2-furancarboxaldehyde-phosphate (4-HFC-P) from glyceraldehyde-3-phosphate catalyzed by 4-HFC-P synthase. Biochemistry 54(19):2997–3008. 10.1021/acs.biochem.5b00176 25905665 10.1021/acs.biochem.5b00176

[CR40] Wang Yu, Huimin Xu, Jones MK, White RH (2015b) Identification of the final two genes functioning in methanofuran biosynthesis in *Methanocaldococcus Jannaschii*. J Bacteriol 197(17):2850–2858. 10.1128/JB.00401-15 26100040 10.1128/JB.00401-15 PMC4524034

[CR41] Wang Yu, Brown CA, Chen R (2018) Industrial production, application, microbial biosynthesis and degradation of furanic compound, hydroxymethylfurfural (HMF). AIMS Microbiology 4(2):261–273. 10.3934/microbiol.2018.2.261 31294214 10.3934/microbiol.2018.2.261 PMC6604932

[CR42] White RH (1988) Structural diversity in the methanofuran from different methanogenic bacteria. J Bacteriol 170(10):4594–4597 3170480 10.1128/jb.170.10.4594-4597.1988 PMC211496

