# Supplementary material for: Molecular basis of hyper-thermostability in the thermophilic archaeal aldolase MfnB
Source: Extremophiles. 2024 Aug 31;28(3):42. doi: 10.1007/s00792-024-01359-x (PMC11365854; doi:10.1007/s00792-024-01359-x)
Supplement: Supplementary file 1 — Supplementary file1 (DOCX 374 KB) [file 792_2024_1359_MOESM1_ESM.docx]

**Supplementary Information**

Molecular Basis of Hyper-thermostability in the Thermophilic Archaeal Aldolase MfnB

Rosie M. A. Maddock, Carl O. Marsh, Samuel T. Johns, Lynden D. Rooms, Phillip W. Duke, Marc W. van der Kamp, James E. M. Stach, Paul R. Race

**Table S1.** Methanogen species and sequences used for phylogenetic analysis.

| Genus | Species | Strain | Gene accession | Protein accession | Thermotolerance | Publication |
| --- | --- | --- | --- | --- | --- | --- |
| *Methanosalsum* | *zhilinae* | DSM 4017 | CP002101 | WP_013897696.1 | mesophile | I. Mathrani et al., 1988, Int. J. Syst. Bacteriol., vol. 38, p. 139 |
| *Methanothermobacter* | *wolfeii* |  | CP044013 |  | extreme thermophile | J. Winter et al., 2000, Int. J. Syst. Evol. Microbiol., vol. 50, p. 43 |
| *Methanobrevibacter* | *woesei* |  | PWB87014 |  | mesophile | T. Miller et al., 2002, Int. J. Syst. Evol. Microbiol., vol. 52, p. 819 |
| *Methanococcus* | *voltae* | A3 | CP002057 |  | mesophile | W. Balch et al., 1979, Microbiol. Rev., vol. 43, p. 260 |
| *Methanocaldococcus* | *villosus* | KIN24-T80 | ENN95838 |  | hyperthermophile | A. Bellack et al., 2011, Int. J. Syst. Evol. Microbiol., vol. 61, p.1239 |
| *Methanococcus* | *vannielii* | SB | CP000742 |  | mesophile | T. Stadtman et al., 1951, J. Bacteriol., vol. 62, p. 269 |
| *Methanosarcina* | *thermophila* |  | AP017646 |  | extreme thermophile | G. Patel et al., 1990, Int. J. Syst. Bacteriol., vol. 40, p. 79 |
| *Methanothrix* | *thermoacetophila* | PT | CP000477 |  | thermophile | N/A |
| *Methanobrevibacter* | *thaueri* |  | PWB87919 |  | mesophile | T. Miller et al., 2002, Int. J. Syst. Evol. Microbiol., vol. 52, p. 819 |
| *Methanothermobacter* | *tenebrarum* |  | RAO79032 | WP_112094009.1 | hyperthermophile | K. Nakamura et al., 2013, Int. J. Syst. Evol. Microbiol., vol. 63, p. 715 |
| *Methanosphaera* | *stadtmanae* |  | LR698975 | WP_112149367.1 | mesophile | T. Miller et al., 1985, Arch. Microbiol., vol. 141, p. 116 |
| *Methanobacterium* | sp. | 42_16 | AIS31913 |  | mesophile | P. Hu et al., 2016, mBio, vol. 7(1), e01669-15 |
| *Methanobacterium* | sp. | PtaB.Bin024 | OPX59439 |  | mesophile | M. Nobu et al., 2017. Environ.Microbiol. Vol. 19, p. 4576 |
| *Methanobacterium* | sp. | PtaU1.Bin097 | OPY24569 |  | mesophile | M. Nobu et al., 2017. Environ.Microbiol. Vol. 19, p. 4576 |
| *Methanobrevibacter* | sp. | AbM4 | CP004050 |  | mesophile | S. Leahy., et al. 2013, Stand Genomic Sci., vol. 8, p. 215 |
| *Methanosphaera* | sp. | SHI613 | RAP51343 |  | mesophile | F. Karlsson et al., 2013, Nature., vol. 498, p. 99 |
| *Methanosphaera* | sp. | rholeuAM130 | RAP54161 |  | mesophile | F. Karlsson et al., 2013, Nature., vol. 498, p. 99 |
| *Methanosphaera* | sp. | BMS | CP014213 |  | mesophile | N/A |
| *Methanosphaera* | sp. | rholeuAM270 | RAP53265 |  | mesophile | F. Karlsson et al., 2013, Nature., vol. 498, p. 99 |
| *Methanosphaera* | sp. | SHI1033 | RAP44721 |  | mesophile | F. Karlsson et al., 2013, Nature., vol. 498, p. 99 |
| *Methanothrix* | *soehngenii* | GP6 | CP002565 |  | mesophile | R. Barber et al., 2011, J. Bacteriol., vol. 193, p. 3668 |
| *Methanobrevibacter* | *smithii* |  | CP017803 |  | mesophile | W. Balch et al., 1979, Microbiol. Rev., vol. 43, p. 260 |
| *Methanoculleus* | *sediminis* |  | KLK87765 |  | thermophile | Sheng-Chung Chen et al., 2015, Int. J. Syst. Evol. Microbiol., vol. 65, p. 2141 |
| *Methanobrevibacter* | *ruminantium* | M1 | CP001719 |  | mesophile | H. Smith et al., 1958, J. Bacteriol., vol. 75, p. 713 |
| *Methanolobus* | *psychrophilus* | R15 | CP003083 | AFV23502.1 | psychrophile | G. Zhang et al., 2008, Appl. Environ. Microbiol., vol. 74, p. 6114 |
| *Methanobacterium* | *paludis* |  | CP002772 |  | mesophile | Cadillo-Quiroz et al., 2014, Int. J. Syst. Evol. Microbiol., vol. 64, p. 1473 |
| *Methanocella* | *paludicola* | SANAE | AP011532 |  | mesophile | S. Sakai et al., 2008, Int. J. Syst. Evol. Microbiol., vol. 58, p. 929 |
| *Methanobrevibacter* | *oralis* |  | KZX11986 |  | mesophile | A. Ferrari et al., 1994, Curr. Microbiol., vol. 29, p. 7 |
| *Methanothermococcus* | *okinawensis* | IH1 | CP002792 |  | extreme thermophile | K. Takai et al., 2002, Int. J. Syst. Evol. Microbiol., vol. 52, p. 1089 |
| *Methanobrevibacter* | *millerae* |  | CP011266 |  | mesophile | S. Rea et al., 2007, Int. J. Syst. Evol. Microbiol., vol. 57, p. 450 |
| *Methanoculleus* | *marisnigri* | JR1 | CP000562 | WP_011844409.1 | mesophile | J. Romesser et al., 1979, Arch. Microbiol., vol. 121, p. 147 |
| *Methanococcus* | *maripaludis* | C7 | CP000745 | A6VK15.1 | mesophile | W. Jones et al., 1983, Arch. Microbiol., vol. 135, p. 91 |
| *Methanococcus* | *maripaludis* | S2 | BX950229 |  | mesophile | W. Jones et al., 1983, Arch. Microbiol., vol. 135, p. 91 |
| *Methanothermobacter* | *marburgensis* | str. Marburg | CP001710 | WP_013295410.1 | extreme thermophile | A. Wasserfallen et al., 2000, Int. J. Syst. Evol. Microbiol., vol. 50, p. 43 |
| *Methanobacterium* | *lacus* |  | CP002551 | WP_013644580.1 | mesophile | G. Borrel et al., 2012, Int. J. Syst. Evol. Microbiol., vol. 62, p. 1625 |
| *Methanocorpusculum* | *labreanum* | Z | CP000559 |  | mesophile | Y. Zhao et al., 1989, Int. J. Syst. Evol. Microbiol., vol. 39, p. 10 |
| *Methanopyrus* | *kandleri* | AV19 | AE009439 | WP_011019970.1 | hyperthermophile | M. Kurr et al., 1991, Arch. Microbiol., vol. 156, p. 239 |
| *Methanocaldococcus* | *jannaschii* | DSM 2661 | L77117 | Q58499.1 | hyperthermophile | W. Jones, et al., 1983, Arch. Microbiol., vol. 136, p. 254 |
| *Methanocaldococcus* | *infernus* | ME | CP002009 |  | hyperthermophile | C. Jeanthon et al., 1998, Int. J. Syst. Bacteriol., vol. 48, p. 913 |
| *Methanotorris* | *igneus* | Kol S | CP002737 |  | hyperthermophile | S. Burggraf et al., 1990, Syst. Appl. Microbiol., vol. 13, p. 263 |
| *Methanotorris* | *formicicus* | Mc-S-70 | EHP89680 |  | hyperthermophile | K. Takai et al., 2004, Int. J. Syst. Evol. Microbiol., vol. 54, p. 1095 |
| *Methanobacterium* | *formicicum* |  | EKF86478 |  | mesophile | M. Bryant et al., 1987, Int. J. Syst. Bacteriol., vol. 37, p. 171 |
| *Methanosarcina* | *flavescens* |  | CP032683 |  | thermophile | T. Kern et al., 2016. Int J Syst Evol Microbiol vol. 66 p. 1533 |
| *Methanothermus* | *fervidus* | DSM 2088 | CP002278 | WP_013413276.1 | hyperthermophile | K. Stetter et al., 1981, Zentralbl. Mikrobiol., vol. 178, p. 166 |
| *Methanocaldococcus* | *fervens* | AG86 | CP001696 |  | hyperthermophile | C. Jeanthon et al., 1999, Int. J. Syst. Bacteriol., vol. 49, p. 583 |
| *Methanohalobium* | *evestigatum* | Z-7303 | CP002069 |  | thermophile | T. Zhilina et al., 1987, Dokl. Akad. Nauk SSSR, vol. 293, p. 464 |
| *Methanobrevibacter* | *cuticularis* |  | KZX14505 |  | mesophile | J. Leadbetter et al., 1996, Appl. Environ. Microbiol., vol. 62, p. 3620 |
| *Methanosphaera* | *cuniculi* |  | PAV07894 |  | mesophile | B. Biavati et al., 1988, Appl. Environ. Microbiol., vol. 54, p. 768 |
| *Methanocella* | *conradii* | HZ254 | CP003243 |  | thermophile | Z. Lu et al., 2012, J. Bacteriol., vol. 194, p. 2398 |
| *Methanobacterium* | *congolense* |  | LT607756 |  | mesophile | N. Cuzin et al., 2001, Int. J. Syst. Evol. Microbiol., vol. 51, p. 489 |
| *Methanoculleus* | *chikugoensis* |  | SCL76371 |  | mesophile | D. Dianou et al., 2001, Int. J. Syst. Evol. Microbiol., vol. 51, p. 1663 |
| *Methanobacterium* | *bryantii* |  | PAV03896 |  | thermophile | W. Balch et al., 1979, Microbiol. Rev., vol. 43, p. 260 |
| *Methanoregula* | *boonei* | 6A8 | CP000780 |  | mesophile | S. Brauer et al., 2011, Int. J. Syst. Evol. Microbiol., vol. 61, p. 45 |
| *Methanocella* | *arvoryzae* | MRE50 | AM114193 | WP_012036435.1 | thermophile | S. Sakai et al., 2010, Int. J. Syst. Evol. Microbiol., vol. 60, p. 2918 |
| *Methanobacteriales* | *archaeon* |  | PGYO01000001 |  | mesophile | A. Hernsdorf et al., 2017, The ISME J, vol. 11, p. 1915 |
| *Methanobrevibacter* | *arboriphilus* |  | OQD58067 |  | mesophile | J. Zeikus et al., 1975, Antonie Van Leeuwenhoek, vol. 41, p. 543 |
| *Methanococcus* | *aeolicus* | Nankai-3 | CP000743 |  | thermophile | M. Kendall et al., 2006, Int. J. Syst. Evol. Microbiol., vol. 57, p. 1525 |

**Figure S1.** Comparative amino acid sequence alignment of MfnB_MJ and MfnB_MM. Conversed residues are shown as white text in red boxes, residues that differ between the two proteins are shown as red text in white boxes. Secondary structure elements identified in the *M. jannaschii* MfnB crystal structure (PDB ID 4RC1) are shown for reference.


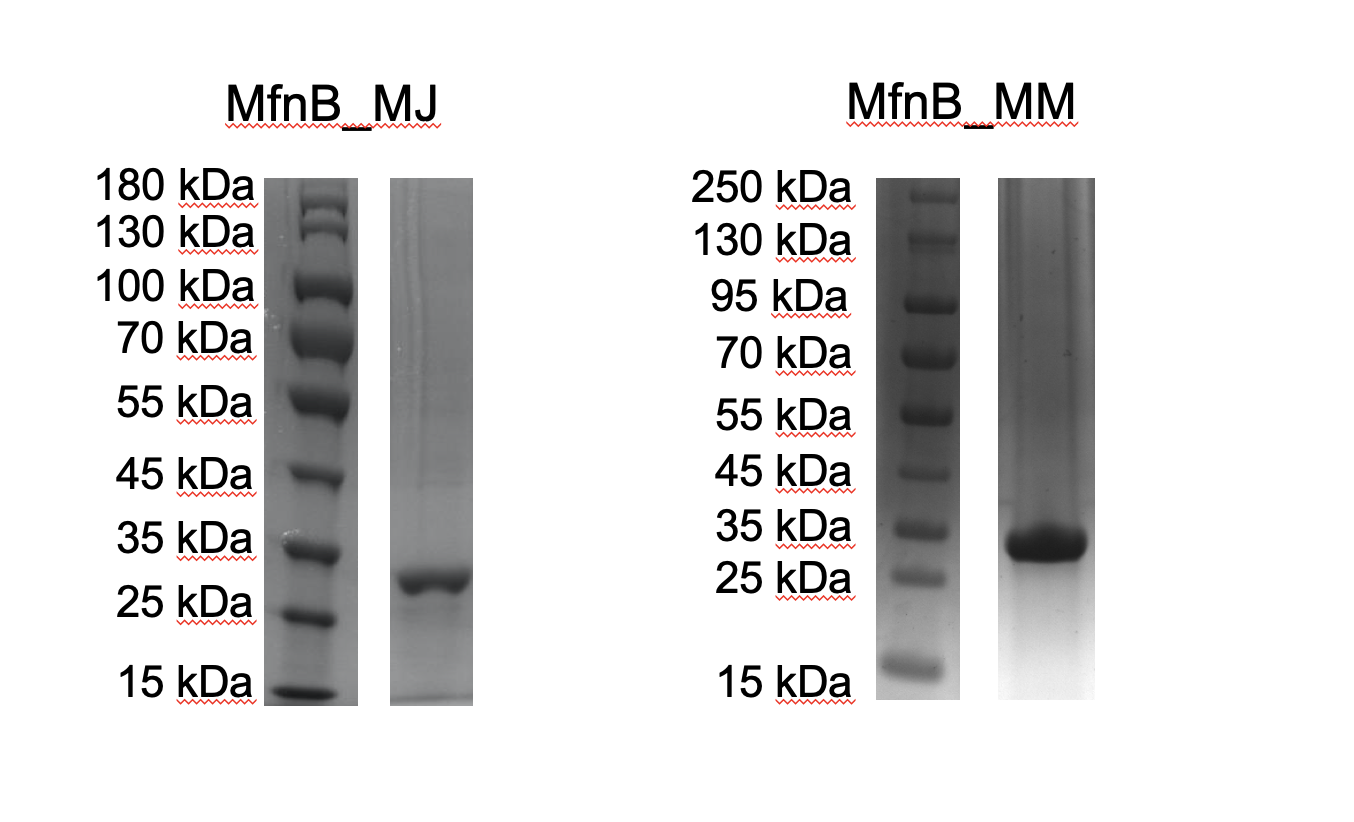


**Figure S2.** SDS-PAGE analysis of purified recombinant MfnB_MJ and MfnB_MM.
